# Root-centered sodium sequestration and transcriptomic regulation under salt and alkali stress in wild soybean (Glycine soja)

**DOI:** 10.3389/fpls.2025.1675559

**Published:** 2025-09-18

**Authors:** Meng Wang, Yingyu Qu, Xueli Lu, Syeda Wajeeha Gillani, Yiru Song, Yu Bai, Yiqiang Li, Chengsheng Zhang, Zongchang Xu, Chen Meng

**Affiliations:** ^1^ College of Agriculture, Qingdao Agricultural University, Qingdao, China; ^2^ Marine Agriculture Research Center, Tobacco Research Institute of Chinese Academy of Agricultural Sciences, Qingdao, China; ^3^ Qingdao Key Laboratory of Coastal Saline-alkali Land Resources Mining and Biological Breeding, Qingdao, China; ^4^ National Center of Technology Innovation for Comprehensive Utilization of Saline-Alkali Land, Dongying, China

**Keywords:** ion homeostasis, flavonoids, abiotic stress, ion compartmentalization, wild soybean

## Abstract

Salt and alkali stress are major constraints on soybean productivity, but their distinct impacts during early development remain insufficiently understood. Wild soybean (*Glycine soja*), a valuable genetic resource for stress tolerance, was evaluated under salt (0.6% and 1.2% NaCl) and alkali (pH 9.16) stress by assessing germination, seedling traits, ion accumulation, and transcriptomic responses. Salt stress permitted partial germination, whereas alkali stress completely suppressed radicle emergence. Seedling growth and height showed tolerance under salinity, but high pH caused severe wilting and mortality. Ion profiling revealed root Na^+^ sequestration with stem K^+^ buffering in salinity, whereas alkali stress confined Na^+^ to roots, maintaining the highest stem K^+^/Na^+^ ratio. Bioaccumulation and translocation factors peaked at 0.6% NaCl in wild soybean. Transcriptome analysis identified 7,355 DEGs grouped into five clusters, enriched in phenylpropanoid/flavonoid biosynthesis and hormone signaling. Salt stress upregulated genes including FLS, F3H, and F3′5′H, whereas alkali stress induced CHS, peroxidase, and CYP75B1. Ion transport regulation differed, with HKT1 and KT11 activated under salinity and NIP5–1 under alkalinity. Among 385 TF-related DEGs, MYB, ERF, bHLH, and WRKY dominated (67% of total), with complex TF-gene networks observed under salt stress. Exogenous flavonoids (rutin, eriodictyol) treatment enhanced leaf area, root length, and plant height under salt and alkali stress treatments. These results suggest that *G. soja* mitigates ion toxicity via root Na^+^ sequestration, stress-responsive gene regulation, and flavonoid-mediated growth enhancement, providing insights into adaptive mechanisms under salt and alkali stress.

## 1 Introduction

Saline-alkaline stress resulting from soil salinization and/or alkalization has emerged as a major global environmental challenge, significantly compromising agricultural productivity and ecological stability (Zhao et al., 2022). According to GSAS map data (https://www.fao.org/global-soil-partnership/gsasmap/en/), over 6% of the world’s soils are already affected by salinity and alkalinity (Cai et al., 2022). Unsustainable irrigation practices and the excessive fertilizers application are further exacerbating land degradation through secondary salinization. It is estimated that approximately 50% of cultivated land may become salinized by 2050 (Wang et al., 2003; Zhou et al., 2023).

Saline-alkali soils are classified into neutral-pH soil, which are rich in NaCl and Na_2_SO_4_ (pH∼7), and high-pH sodic soils, characterized by high concentrations of Na_2_CO_3_ or NaHCO_3_ (pH>8.5) (Hou et al., 2023).While salt stress in soil typically induces osmotic stress and ion toxicity to plant tissues, alkaline stress imposes an additional detriment due to elevated pH, rendering it more severe than salt stress alone (Fang et al., 2021; Rao et al., 2023). The high concentrations of carbonates (CO_3_^2−^) and bicarbonates (HCO_3_^−^) ions in alkaline soils lead to alkaline soil pH and reduced plant nutrients availability (Sagervanshi et al., 2022). Unlike neutral saline soils, high-pH saline-alkaline soils further inhibit the uptake of essential nutrients and sodium ion (Na^+^) exclusion (Zhang et al., 2023). In addition, high alkalinity stress induces excessive reactive oxygen species (ROS) accumulation, causing oxidative damage to cellular proteins, lipids, and DNA, and potentially leading to cell apoptosis (Javid et al., 2012; Sun et al., 2016; Zhang et al., 2017; An et al., 2020).

Cultivated soybean (*Glycine max* L.) was domesticated from its wild progenitor *Glycine soja*. During this domestication process, only approximately 50% of the genes present in wild soybean were retained, resulting in the loss of numerous stress-adaptive genes (Kofsky et al., 2018). Consequently, wild soybean represents a valuable genetic resource for enhancing salt-alkali tolerance in cultivated varieties. Studies have shown that wild soybean maintains higher genetic diversity and superior adaptability to abiotic stresses compared to cultivated varieties (Ge et al., 2010; Cai et al., 2022). For instance, under salt and alkali stress, wild soybean accumulates higher concentrations of osmo-protectants such as proline and glycine betaine, which contribute to maintaining osmotic balance and protecting cellular structures protection (Wu et al., 2014). Furthermore, wild soybean exhibits notably lower Na^+^ accumulation, higher K^+^ retention, and consequently a higher K^+^/Na^+^ ratio in both leaves and roots compared to salt-sensitive cultivated soybean germplasms under salt stress (Liu et al., 2017).

Nonetheless, prior research on wild soybean have focused exclusively on its response to either salt stress or alkali stress alone. Consequently, research directly comparing the physiological and molecular responses of wild soybean to both stresses remains limited (Sun et al., 2025). Identifying stress resistance genes in wild soybean and utilizing them into cultivated germplasm could enhance stress resistance and overall crop quality, while also addressing the issues of limited genetic diversity and inadequate environmental adaptability (Xie et al., 2019).The objective of this work is to (i) examine the responses of wild soybean to both saline and alkaline stresses at physiological, biochemical, and molecular levels, and (ii) investigate the mechanisms underlying its tolerance to these stresses. This work will not only contribute to the improvement of salt and alkali tolerance in soybean breeding but also offers significant insights into the broader molecular mechanisms underlying plant stress responses and environmental adaptability.

## 2 Materials and methods

### 2.1 Plant materials

Wild soybean seeds were collected from Dongying City (37°17053.4500 N, 118°37015.0400 E), Shandong Province, China. The seeds were the self-pollinated progeny of a single wild soybean plant and were stored under laboratory conditions for experimental use (Xu et al., 2020b).

### 2.2 Seeds pre-treatment, stress application, and plant growth conditions

To enhance the germination rate of wild soybean seeds, the seeds were soaked in concentrated sulfuric acid and repeatedly agitated to disrupt the mud coating. Following treatment, the seeds were thoroughly rinsed three times with deionized water and subsequently used for experimental purposes. Salt stress treatments was treated were administered using 0.6% and 1.2% NaCl solutions prepared in 1/20 strength Murashige and Skoog (MS) medium. Alkaline stress treatments were applied using Na_2_CO_3_-NaHCO_3_ buffer solution (also in 1/20 MS) at pH values of 9.16 (0.1M Na_2_CO_3_:0.1M NaHCO_3_ = 1:9), 9.90 (0.1M Na_2_CO_3_:0.1M NaHCO_3_ = 5:5) and 10.83 (0.1M Na_2_CO_3_:0.1M NaHCO_3_ = 8:2). A 1/20 MS solution without NaCl or alkali buffer served as the control.

For the growth experiment under salt and alkaline stress, seeds were sown in vermiculite and irrigated with 1/20 MS solution. After three weeks, uniform seedlings were exposed to the respective salt and alkali treatments. Each treatment comprised 32 seedlings per replicate, with three replicates per treatment. Photographs of the aerial portions were taken on the third and seventh days after treatment. After 7 days of treatments, root, stem, and leaf tissues were harvested, immediately immersed in liquid nitrogen, and stored in -80°C for subsequent physiological, biochemical, and transcriptomic analyses. Seed germination and plant growth conditions were consistent to previous protocols (Xu et al., 2020b), including a 16:8 h light:dark photoperiod, a temperature range of 20 ***-*** 22°C, and relative humidity of 65 ***–*** 75%.

### 2.3 Sodium and potassium content measurement

The concentrations of Na^+^ and K^+^ were determined following a modified protocol from a previous study (Xu et al., 2020a). In summary, 0.25 g of oven-dried and powdered plant tissues were digested in 5 mL of HNO_3_ at 110°C for approximately 6 hours until a clear, colorless solution was achieved. After cooling, the digested samples were diluted to a volume of 10 mL using deionized water. Na^+^ and K^+^ contents were measured using an optical emission spectrometer (ICP-OES, Optima 8000, PerkinElmer, USA).

### 2.4 Bioaccumulation factor and translocation factor calculation

The bioaccumulation factor (BF), representing the ability of plant to uptake and retain ions, was determined based on previously described methods with minor modifications. BF was calculated as the ratio of Na^+^ content in plant tissue (root, stem, and leaf; mg kg^-1^ dry matter) to) to its corresponding concentration in the soil (Yoon et al., 2006). The translocation factor (TF), indicating the efficiency of ion transport from roots to shoots, was calculated as the ratio of Na^+^ content in the shoots to that in the roots, both expressed on a dry matter basis (mg kg^-1^) (Padmavathiamma and Li, 2007; Zhou et al., 2013).

### 2.5 Transcriptome sequencing and KEGG enrichment analysis

Total RNA was extracted from root tissues of seedlings exposed to salt stress (0.6% NaCl and 1.2% NaCl), alkali stress (pH=9.16), and control conditions using an EasyPure^®^plant RNA kit (ER301-01, TransGen Biotech). RNA quality was assessed using 1% agarose gel, a 2100 Bioanalyzer (Agilent Technologies, USA), and a NanoDrop2000 spectrophotometer (Thermo FisherScientific, USA). High-quality RNA was used for library construction and sequencing, which was performed by Shanghai Majorbio Bio-Pharm Biotechnology Co., Ltd. (Shanghai, China). Raw reads were filtered using FASTP (https://github.com/OpenGene/fastp/), and clean reads were aligned to the reference genome using while HISAT2 (http://ccb.jhu.edu/software/hisat2/). Transcript assembly was carried out with StringTie (https://ccb.jhu.edu/software/stringtie/). Each treatment was represented by three biological replicates. Transcript expression levels were quantified using the transcripts per million reads (TPM) method. Differentially expressed genes (DEGs) were identified using DESeq2, DEGseq, edgeR, Limma, or NOIseq tools (threshold: *P*-adjust ≤ 0.05 and |log_2_ fold change| ≥ 1). Gene ontology(GO) and Kyoto Encyclopedia of Genes and Genomes (KEGG) pathway enrichment analysis was conducted using the KOBAS database (http://bioinfo.org/kobas) to elucidate relevant metabolic pathways and biological functions associated with DEGs.

### 2.6 Flavonoids supplementation experiment

Wild soybean seedlings were cultured as described above. Half of the three-days-old seedlings received 100 μM of flavonoids rutin and eriodictyol for three days, then all the seedlings were treated with 0.6% NaCl, 1.2% NaCl, or a Na_2_CO_3_-NaHCO_3_ buffer solution (pH=9.16), while seedlings irrigated with 1/20 MS solution served as the control. Five days after stress exposure, plant height, root length, and leaf area were measured in all groups.

### 2.7 RNA extraction and reverse transcription quantitative PCR

Total RNA was isolated from the samples using RNA Kit reagent (Vazyme Biotech, China) following the manufacturer’s instructions. First-strand cDNA was synthesized using a HiScript II 1st Strand cDNA Synthesis Kit (Vazyme Biotech, China) according to the manufacturer’s protocol. RT-qPCR was performed using ChamQ Universal SYBR qPCR Master Mix (Vazyme Biotech). *GsGAPDH* was used as a housekeeping gene. Each sample was analyzed in three biological replicates, and relative expression levels were obtained using the 2^−△△Ct^ method.

### 2.8 Data analysis

All data were subjected to one-way analysis of variance (ANOVA), and means were compared using Tukey’s *post hoc* test at *P* < 0.05 level using SPSS software v 17.0 (SPSS Inc., Chicago, IL, USA).

## 3 Results

### 3.1 Salt and alkali stress inhibit germination and seedling development of wild soybean

**Figure 1:**
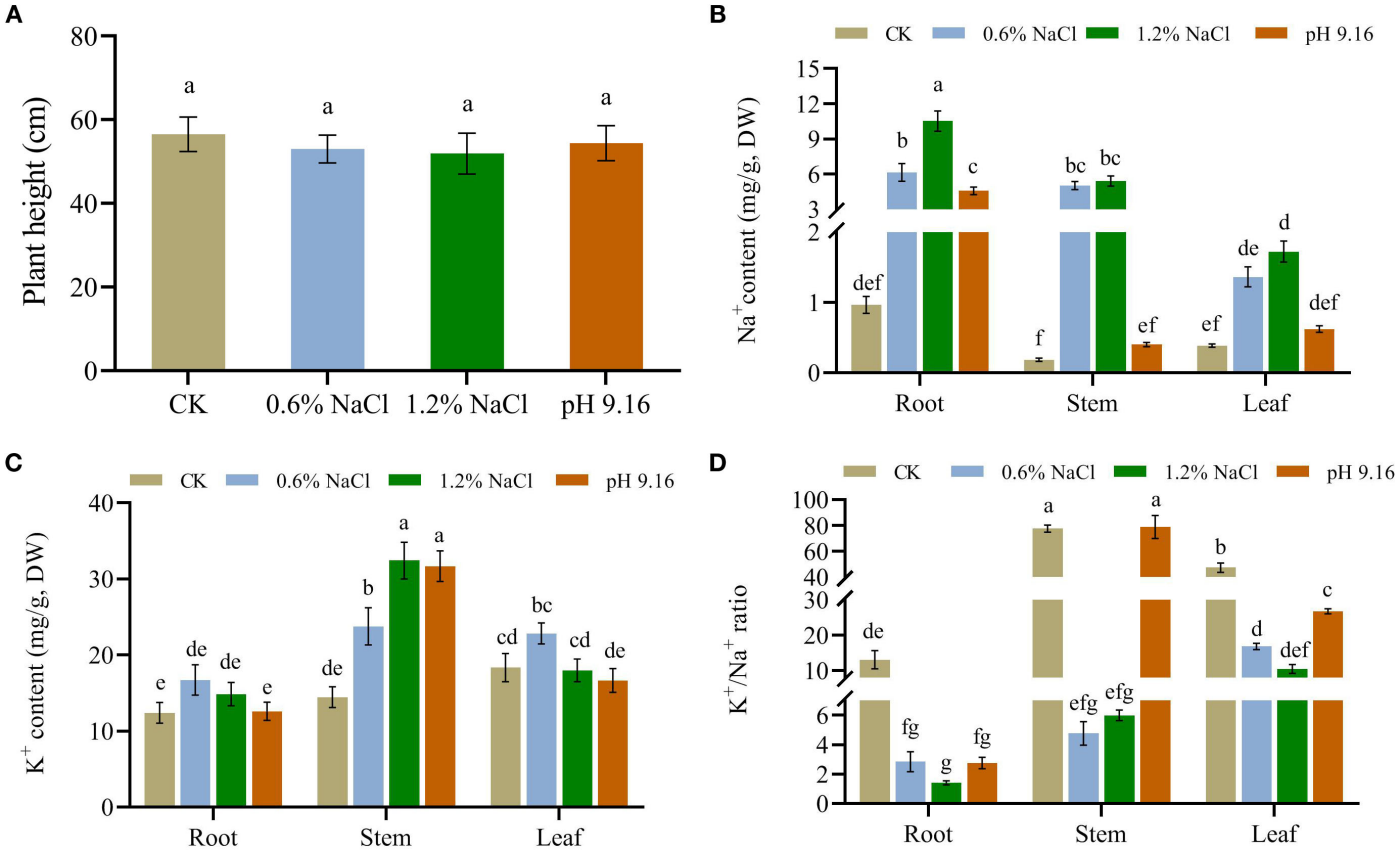
Plant height and ion distribution in wild soybean under salt and alkali stress. **(A)** Plant height; **(B)** Na^+^ content; **(C)** K^+^ content; and **(D)** K^+^/Na^+^ ratio in different tissues of wild soybean subjected to control (CK), salt (0.6% and 1.2% NaCl), and alkali (pH 9.16) treatments. Different letters above the bars represent significant differences among treatments (ANOVA and Tukey’s *post hoc* test, *P* < 0.05).

Salt and alkali stress significantly inhibited the germination and early development of wild soybean (*G. soja*). Notably, alkali stress exerted a greater inhibitory effect on seed germination than salt stress. At pH 10.83, no discernible indications of seed germination were observed. Under milder alkali treatments (pH 9.9 and pH 9.16), radicle emergence was limited to penetration of the seed coat, whereas control seeds developed fully extended radicles and elongated cotyledons. In contrast, seeds subjected to 0.6% and 1.2% NaCl showed radical emergence, indicating milder inhibition compared to alkali stress still reduced development relative to the control. Germination performance under 0.6% salt stress was significantly better than that under 1.2% salt stress (**Supplementary Figure 1**). At the seedling stage, wild soybean exhibited improved stress resistance compared to the germination stage. No apparent differences were observed among seedlings treated with salt (0.6% and 1.2% NaCl) and alkali (pH=9.16) stress when compared to the control. However, seedlings treated with pH 10.83 displayed severe wilting within 3 days approached complete mortality by day 7 (**Supplementary Figure 2**). After 7 days of stress exposure, measurements of plant height showed a slight reduction in treated plants compared to the control; however, the differences were not statistically significant (**Figure 1A**). These findings indicate that short-term salt and alkali stress had minimal impact on vertical growth during the early seedling stage.

### 3.2 Root is the primary sodium-accumulating organ in wild soybean

Ion profiling under salt stress revealed a progressive increase in Na^+^ content from roots to leaves, with the highest accumulation observed in roots at 1.2% NaCl. In contrast, Na^+^ under alkali stress was predominantly confined to roots (**Figure 1B**). K^+^ was primarily concentrated in stems under all salt stress levels, with the highest levels detected in stems and leaves at 1.2% NaCl, followed by alkali stress (pH 9.16) in stems (**Figure 1C**). The K^+^/Na^+^ ratio was significantly reduced in all tissues under salt stress, particularly in roots, but showed a moderate increase toward stems and leaves. Under alkali stress, stems maintained the highest K^+^/Na^+^ ratios among all tissues, suggesting their critical role in ionic homeostasis(**Figure 1D**). These results suggest that wild soybean mitigates ion toxicity through root-localized Na^+^ retention and stem-based K^+^ buffering mechanisms.

Consistent with tissue ion content, the bioaccumulation factor (BF) was highest in roots across all treatments, exceeding a value of 1.44 under 0.6% NaCl, 1.23 under 1.2% NaCl, and 1.00 under alkaline conditions (pH 9.16). The maximum BF was observed in roots under 0.6% NaCl. Additionally, the stem BF under 0.6% NaCl also exceeded 1 (1.18). The highest translocation factor (TF) was recorded in plants subjected to 0.6% NaCl; however, TF values remained below 1 across all treatments, with the lowest observed under alkali stress. These results further indicate that roots function as the primary site of Na^+^ accumulation in wild soybean (**Table 1**).

**Table 1:** Bioaccumulation and translocation in root, stem, and leaf tissues of wild soybean under control, salt, and alkali stress conditions.

Treatment	Tissue	Bioaccumulation (BF)	Translocation (TF)
Control	Root	–	–
	Stem	–	0.19 ± 0.01 ^de^
	Leave	–	0.40 ± 0.02 ^c^
0.6% NaCl	Root	1.44 ± 0.11 ^a^	–
	Stem	1.18 ± 0.08 ^b^	0.82 ± 0.03 ^a^
	Leave	0.31 ± 0.01 ^e^	0.22 ± 0.01 ^d^
1.2% NaCl	Root	1.23 ± 0.07 ^b^	–
	Stem	0.63 ± 0.02 ^d^	0.51 ± 0.02 ^b^
	Leave	0.20 ± 0.01 ^ef^	0.16 ± 0.01 ^ef^
pH 9.16	Root	1.00 ± 0.07 ^c^	–
	Stem	0.09 ± ND ^f^	0.09 ± ND ^g^
	Leave	0.13 ± 0.01 ^f^	0.13 ± 0.01^fg^

Values are the means of three replicates ± SD. Values within a column followed by different lowercase letters are significant different different (ANOVA and Tukey’s *post hoc* test, *P < 0.05*).ND, not detected.

### 3.3 Genes enriched in wild soybean under salt and alkali stress

**Figure 2:**
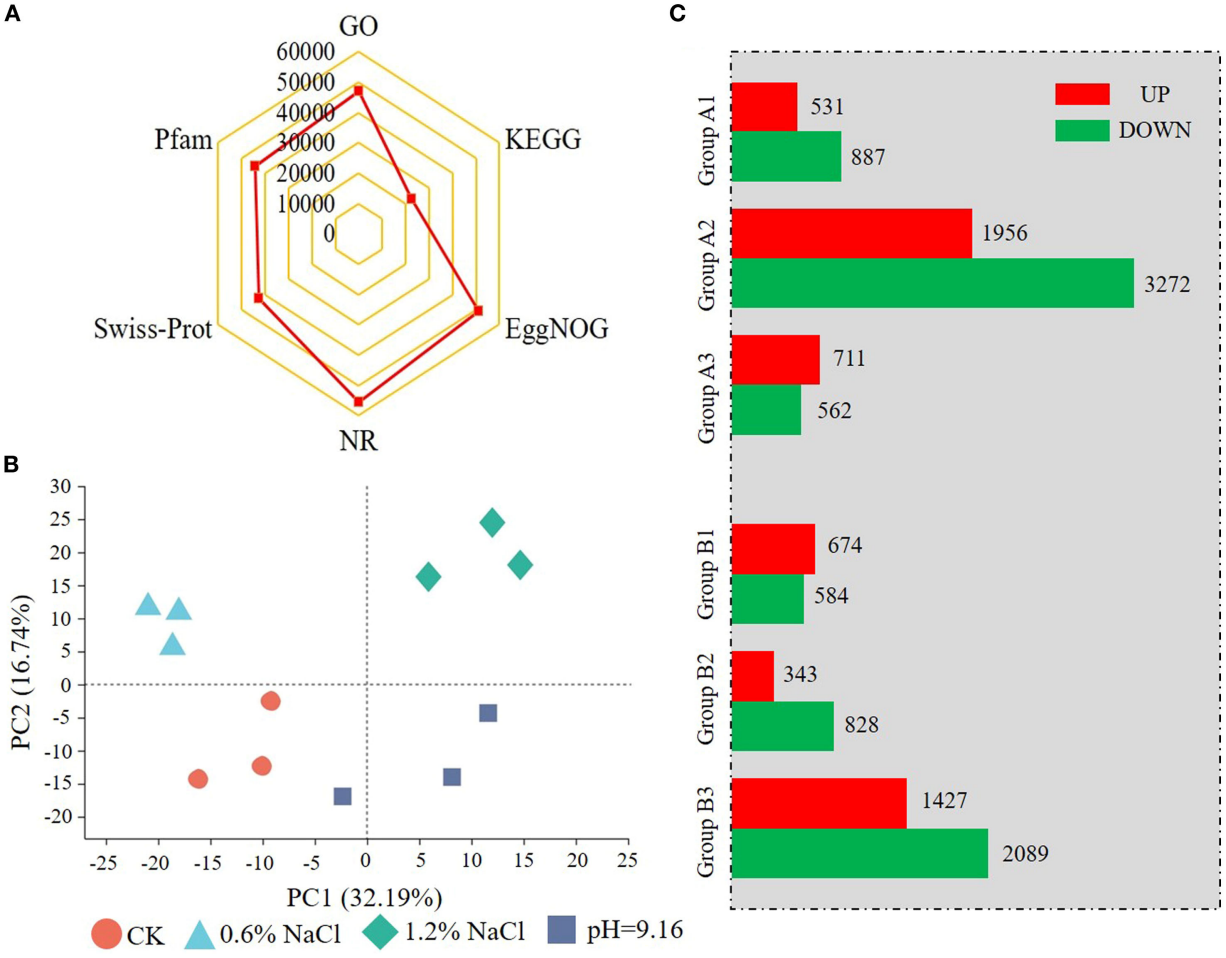
Principal component analysis (PCA), gene annotation statistics **(A, B)**, and differentially expressed gene (DEG) statistics **(C)** of wild soybean under salt and alkali stress. In **(C)** DEGs were identified using thresholds of P < 0.05 and |fold change| ≥ 2. Comparisons include: A1 (0.6% NaCl vs CK), A2 (1.2% NaCl vs CK), A3 (1.2% NaCl vs 0.6% NaCl), B1 (pH 9.16 vs CK), B2 (pH 9.16 vs 0.6% NaCl), and B3 (pH 9.16 vs 1.2% NaCl).

Since root was the primary Na^+^ storage organ, transcriptome sequencing was performed on root samples under salt, alkali, and control conditions. A total of 499,356,810 clean reads (73.38 Gb) were generated. The Q30 score exceeded 93.00% and GC content averaged ~44.00%. Over 92.00% of clean reads uniquely mapped to the *G. soja* reference genome (https://datahub.wildsoydb.org/). The raw sequence data reported in this paper have been deposited in the Genome Sequence Archive (Genomics, Proteomics & Bioinformatics 2021) in National Genomics Data Center (Nucleic Acids Res 2025), China National Center for Bioinformation/Beijing Institute of Genomics, Chinese Academy of Sciences (GSA: CRA028818) that are publicly accessible at https://ngdc.cncb.ac.cn/gsa. Ultimately, about 42,000 unigenes were identified in each sample (**Table 2**), resulting in a total of 55,539 non-redundant unigenes (**Supplementary Table 1**). Among them, 55,534 were in at least one database, with the NR database showing the highest annotation rate (55,531) (**Figure 2A**; **Supplementary Table 1**). Principal component analysis (PCA) showed clear transcriptomic separation across all samples (**Figure 2B**). The samples subjected to alkali stress treatment clustered more closely to the control compared to those under salt stress, whereas the 1.2% NaCl treatment was distinctly separated from all other samples, indicating a greater divergence in response.

**Table 2:** Expression level and annotation of unigenes identified in all sample libraries.

Sample	Clean reads	Clean bases	Total mapped	Q30	GC content	Uniquely mapped	CDS	3’UTR	5’UTR	Unigene NO.
	Sum of three libraries repeat		Mean of the three libraries repeat (%)							
CK	128,416,644	18,784,519,994	63.38	93.39	44.42	91.97	84.4	9.33	2.84	42,680
0.6% NaCl	122,746,114	18,060,020,919	95.02	93.04	44.22	92.49	84.26	8.32	3.43	42,197
1.2% NaCl	124,004,136	18,327,274,662	94.76	93.44	44.04	92.08	83.83	8.09	3.73	41,994
pH-9.16	124,189,916	18,210,795,123	94.16	93.44	43.77	91.06	83.75	8.17	3.66	42.483

DEGs were identified across six comparison groups. Group A consisted of comparisons among salt treatments and control, CK versus 0.6% NaCl (Group A1), 1.2% NaCl (Group A2), and 1.2% NaCl versus 0.6% NaCl (Group A3). While Group B compared alkali stress (pH 9.16) against CK (Group B1), 0.6% NaCl (Group B2), and 1.2% NaCl (Group B3). Group A2 had the highest number of DEGs (5,228), with 1,956 upregulated and 3,272 downregulated DEGs. Group B3 followed with 3,516 DEGs, including 1,427 up-regulated and 2,089 down-regulated (**Figure 2C**; **Supplementary Table 2**). These results indicate that high salt stress (1.2% NaCl) strongly alters gene expression in wild soybean.

### 3.4 DEGs clusters obtained from hierarchical clustering

**Figure 3:**
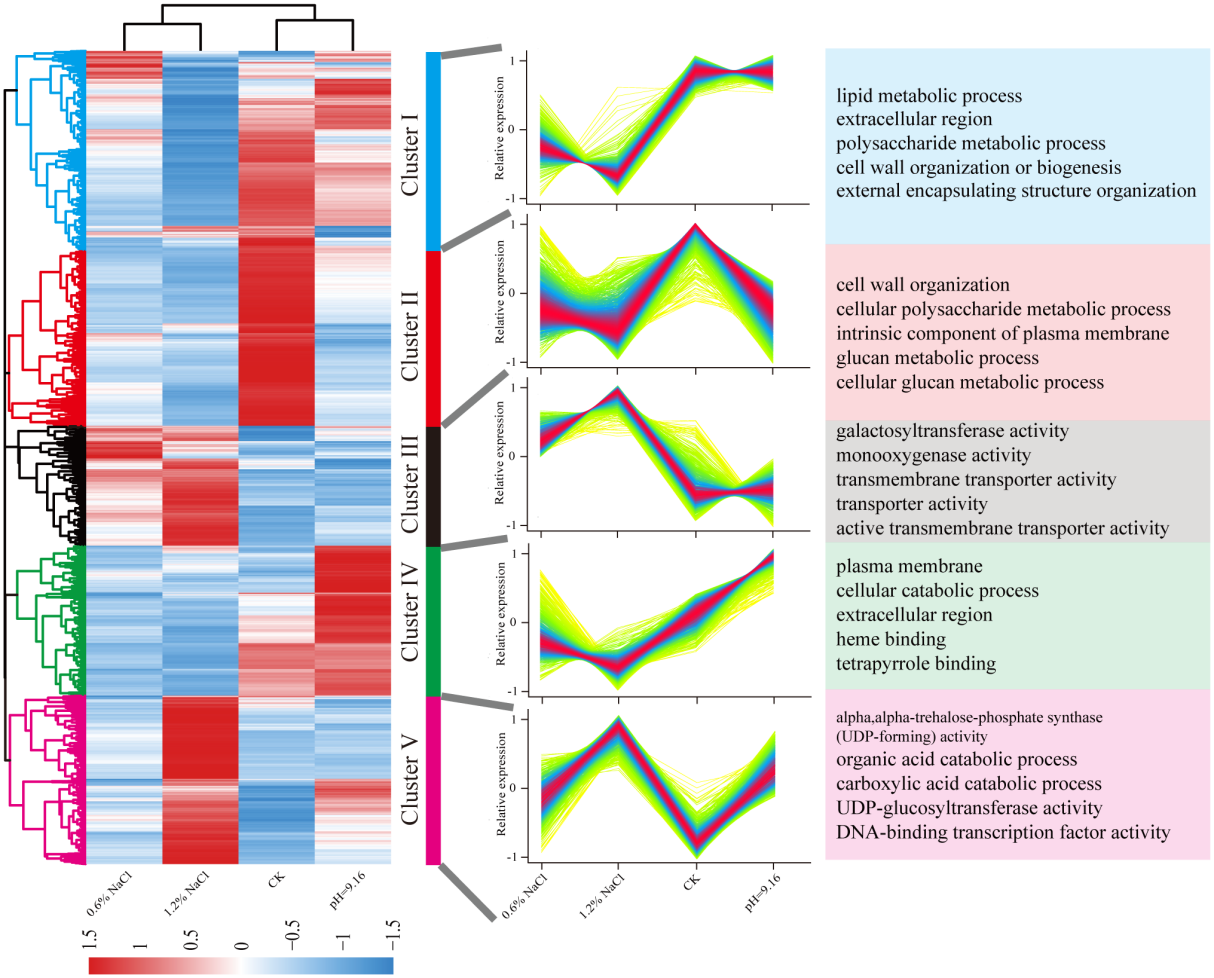
Hierarchical clustering of differentially expressed genes based on normalized TPM values. Heatmap color gradients indicate expression levels, and subclusters represent gene groups enriched in specific GO terms. DEGs expression is visualized as heatmap based on log_2_(fold change), with red and blue indicating high and low expression, respectively.

A total of 7,355 non-redundant DEGs were identified across all comparison groups. Hierarchical clustering categorized these DEGs into five distinct expression clusters (**Figure 3**). Cluster I and IV were down-regulated under salt stress compared to both control and alkali stress. GO enrichment revealed that Cluster I was enriched in “lipid metabolic process” and Cluster IV in “plasma membrane”. Conversely, DEGs in Cluster III were up-regulated under salt stress relative to control. Enriched GO terms included “galactosyltransferase activity”, “transmembrane transporter activity”, and “transporter activity”, indicating involvement of transmembrane transporters and antioxidant-related enzymes in salt stress adaptation. Cluster II displayed down-regulation under both salt and alkali stress compared to the control, with “cell wall organization” as the most enriched term. Cluster V showed exclusive and markedly elevated expression under 1.2% NaCl treatment, with a secondary response observed under 0.6% NaCl and alkali stress treatments. In conclusion, DEGs in Clusters IV were exclusively upregulated under alkali stress but downregulated under salt stress, whereas DEGs in Cluster I and III were specifically different expressed under salt stress. Accordingly, Clusters I, III, and IV were selected for further analysis and downstream investigation.

### 3.5 DEGs enriched in metabolic pathways under salt and alkali stress

**Figure 4:**
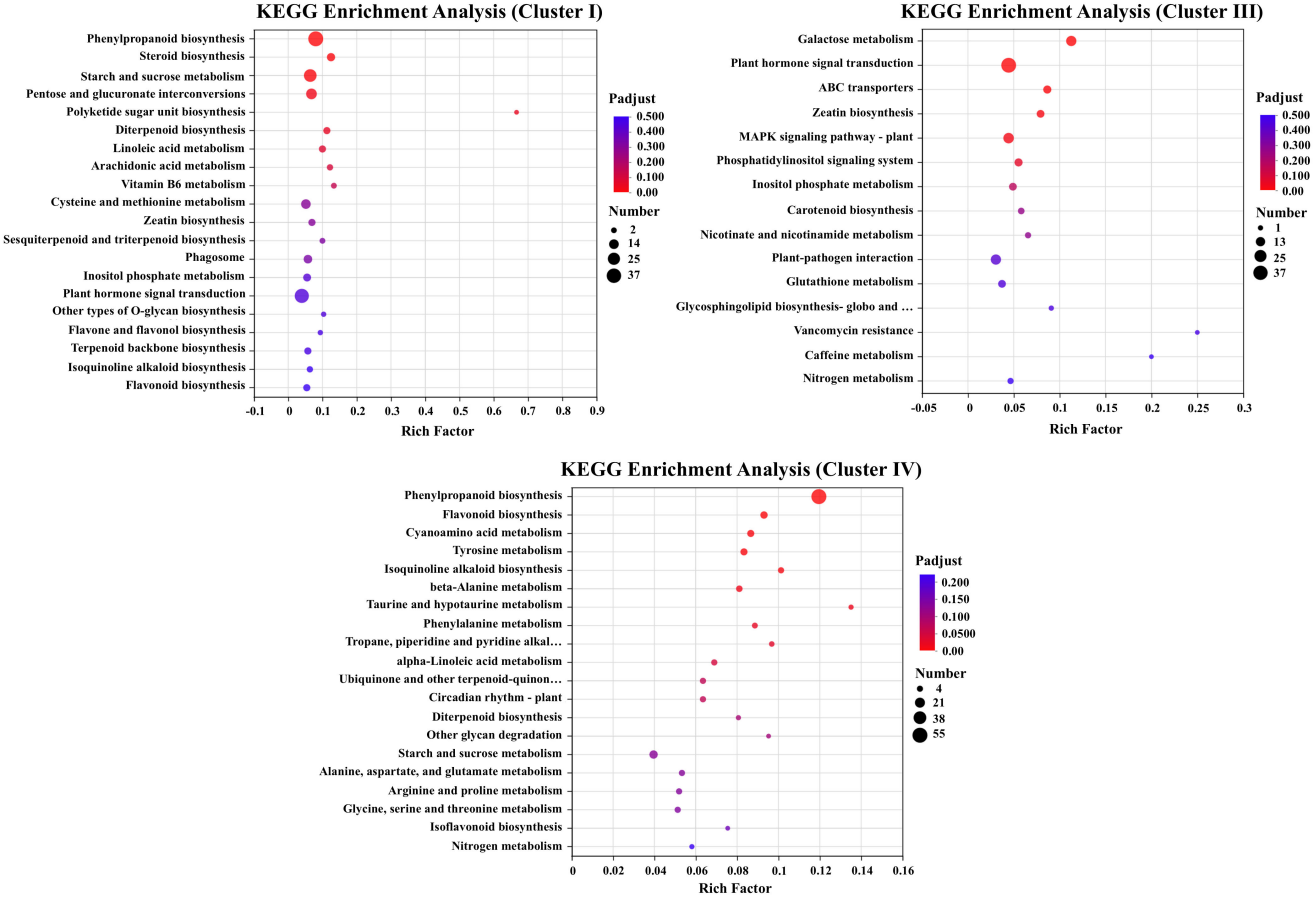
KEGG pathway enrichment analysis of DEGs in Cluster I, Cluster III, and Cluster IV, highlighting significantly enriched biological pathways under salt and alkali stress.

GO enrichment analysis showed that DEGs in Clusters I and IV exhibited similar expression patterns, while DEGs in Cluster III displayed distinct expression profiles under salt stress treatments. The DEGs in Cluster IV were upregulated under alkali stress treatment. KEGG enrichment of the Clusters I (specifically downregulated under salt stress) and Cluster IV(downregulated under salt stress and upregulated under alkali stress) revealed pathways associated with flavonoid metabolism, including “phenylpropanoid biosynthesis” and “flavonoid biosynthesis”. The KEGG of Cluster III (upregulated under salt stress) were enriched in the pathways including “Galactose metabolism”, “Plant hormone signal transduction” and “ABC transporters” (**Figure 4**).

### 3.6 Differential expression of phenylpropanoid and flavonoid biosynthesis genes

**Figure 5:**
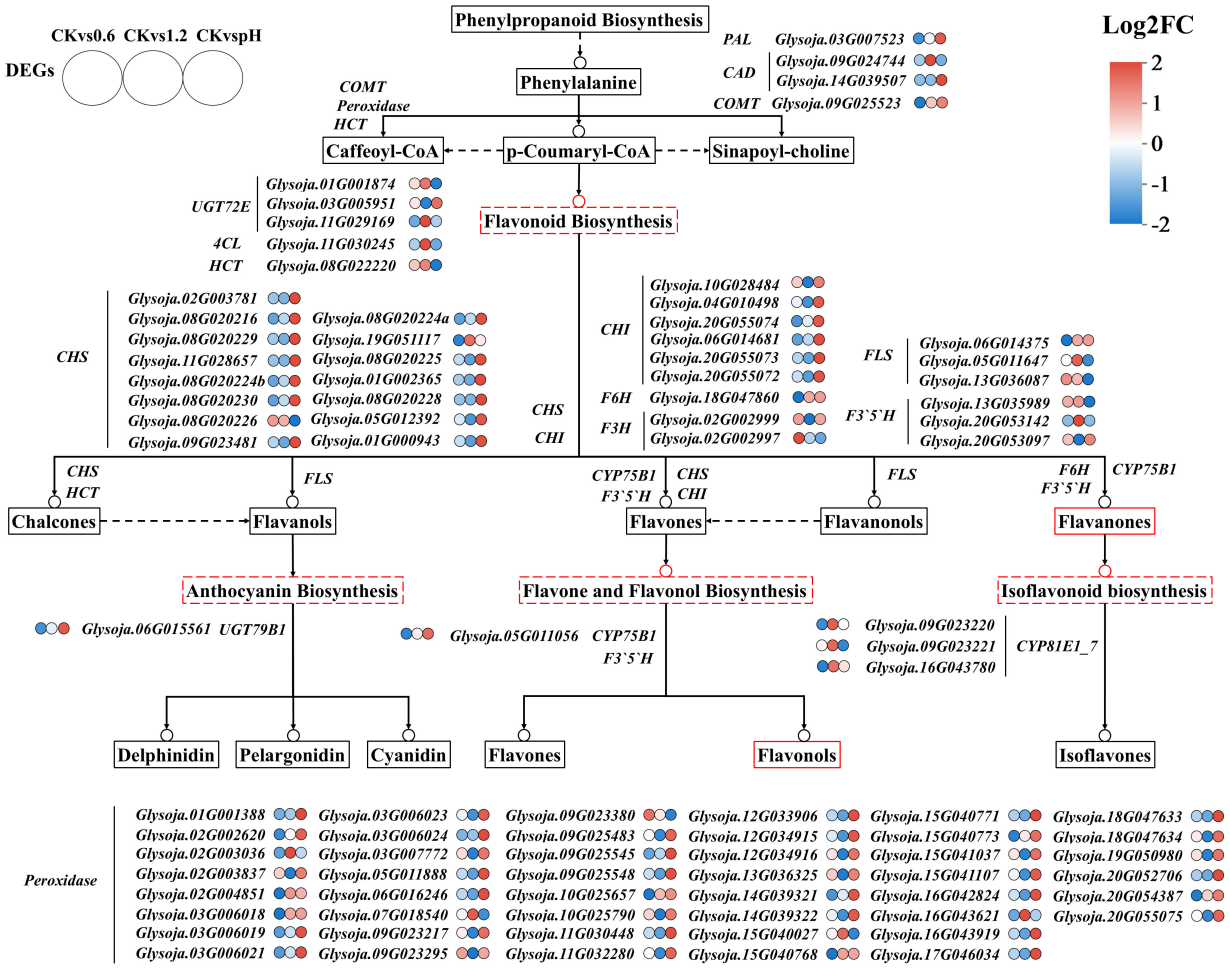
Gene expression profiles of the DEGs annotated to phenylpropanoid and flavonoid biosynthesis pathways in wild soybean under salt and alkali stress. Heatmap shows log_2_(fold change), with red and blue indicating high and low expression, respectively. Key enzymes include *PAL*, phenylalanine ammonia lyase; *4CL*, 4-coumaroyl CoA ligase; *HCT*, shikimate O-hydroxycinnamoyltransferase; *CAD*, cinnamyl-alcohol dehydrogenase; *COMT*, caffeic acid 3-*O*-methyltransferase; *UGT72E*, coniferyl-alcohol glucosyltransferase; *F6H*, flavonoid 6-hydroxylase; *F3H*, flavanone 3 -hydroxylase; *F3’5’H*, flavonoid 3’,5’-hydroxylase; *CHS*, chalcone isomerase; *CHI*, chalcone isomerase; *FLS*, flavonol synthase; *CYP81E*, isoflavone 2’-and 3’-hydroxylases.

To further elucidate the differential metabolic responses, we analyzed expression changes in structural genes within phenylpropanoid and flavonoid biosynthesis pathways. Under alkali stress, a *phenylalanine ammonialyase* (*PAL*) gene and 30 peroxidase genes were specifically upregulated. Moreover, under alkali stress, higher expression was observed for 13 chalcone synthase (*CHS*) genes and 5 chalcone isomerase (*CHI*) genes. In addition, one UDP-glycosyltransferase 79B1 (*UGT79B1*) and one flavonoid 3’-monooxygenase (*CYP75B1*) were exclusively regulated under this treatment. In contrary, two flavone synthase (*FLS*) genes and flavonoid 3’,5’-hydroxylase (*F3’5’H*) were specifically induced under salt stress. These results suggest that flavonoids may play a more critical role in alkali stress adaptation in wild soybean (**Figure 5**; **Supplementary Table 3**).

### 3.7 Sodium transport-related regulatory network under salt and alkali stress

In this study, we identified 29 DEGs associated with Na^+^ influx and storage, most of which were upregulated under salt stress, particularly in the 1.2% NaCl vs. CK comparison group (**Table 3**). This differential expression correlated with observed Na^+^ accumulation levels. Notably, *Glysoja.03G007427* (encoding Sodium transporter HKT1) and *Glysoja.15G041067* (encoding potassium transporter KT11) were specifically upregulated under salt stress. Conversely, *Glysoja.20G054400* and *Glysoja.10G027804* (encoding aquaporin NIP5-1) responded specifically to alkali stress (**Table 3**).

**Table 3:** Differentially expressed genes related to Na^+^ influx and storage across all sample comparisons, including log_2_ fold change, gene description, and gene ID.

Gene_id	Log2 (fold change)						Gene_description
	0.6-G vs CK-G	1.2-G vs CK-G	1.2-G vs 0.6-G	pH-G vs CK-G	pH-G vs 0.6-G	pH-G vs 1.2-G	
*Glysoja.19G052604*	–	1.2128	1.5423	1.4514	1.7866	–	Cyclic nucleotide-gated ion channel 1
*Glysoja.19G052602*	–	–	–	1.0178	–	–	Cyclic nucleotide-gated ion channel 1 isoform B
*Glysoja.04G009075*	–	2.0551	1.3748	–	–	-1.2487	Cyclic nucleotide-gated ion channel 14
*Glysoja.06G015545*	–	-5.2000	–	3.2463	3.3202	8.4587	Glutamate receptor 2.1
*Glysoja.17G045108*	–	3.1023	1.5008	2.8872	1.2924	–	Glutamate receptor 2.5
*Glysoja.16G042877*	–	–	–	1.5342	–	–	Glutamate receptor 2.5 isoform A
*Glysoja.11G031574*	–	1.8005	1.5570	1.3559	1.1168	–	Glutamate receptor 2.7
*Glysoja.14G038063*	–	–	-1.1468	1.8516	1.6299	2.7800	Glutamate receptor 2.7
*Glysoja.11G031577*	–	1.1475	–	–	–	–	Glutamate receptor 2.7 isoform A
*Glysoja.04G010835*	6.2157	5.4829	–	5.3289	–	–	Glutamate receptor 3.2 isoform A
*Glysoja.15G041067*	1.0998	1.3032	–	–	–	–	Potassium transporter 11
*Glysoja.02G004018*	7.4978	7.9216	–	–	-3.9240	-4.3497	Potassium transporter 5
*Glysoja.03G008317*	–	–	–	1.1909	–	–	Potassium transporter 5 isoform A
*Glysoja.20G054400*	–	–	–	1.0291	–	–	Aquaporin NIP5–1 isoform A
*Glysoja.10G027804*	–	–	–	1.2483	–	1.3725	Aquaporin NIP5–1 isoform B
*Glysoja.09G025101*	–	–	-1.0181	–	–	–	Aquaporin NIP2–1 [Glycine soja]
*Glysoja.05G012919*	-1.9161	-2.9214	-1.0004	–	1.4564	2.4597	Aquaporin PIP1–2 isoform A
*Glysoja.08G019327*	-1.4417	-1.9935	–	-1.4287	–	–	Aquaporin PIP1–2 [Glycine soja]
*Glysoja.03G007427*	–	1.3213	–	–	–	–	Cation/H(+) antiporter 20
*Glysoja.08G019902*	3.1030	3.2524	–	–	-5.0605	-5.1977	Vacuolar iron transporter-like 4
*Glysoja.05G012078*	4.3354	4.4384	–	–	-3.0234	-3.1270	Vacuolar iron transporter-like 4
*Glysoja.17G046668*	–	3.0335	–	–	–	-3.2012	Vacuolar membrane protein isoform A
*Glysoja.15G041045*	–	1.1600	–	–	–	–	Vacuolar-sorting receptor 6 isoform B
*Glysoja.01G000023*	–	2.7575	–	–	–	-2.3730	Sodium transporter HKT1 isoform A
*Glysoja.14G038690*	–	3.3939	–	–	–	–	ATPase 7, plasma membrane-type
*Glysoja.13G035921*	–	1.3790	–	–	–	-1.0202	Plasma membrane ATPase 1
*Glysoja.04G009062*	–	1.6337	–	–	–	-1.9368	Plasma membrane ATPase 4 isoform A
*Glysoja.06G014039*	–	1.1074	–	–	–	-1.3425	Plasma membrane ATPase 4 isoform A
*Glysoja.11G031950*	–	–	–	1.9088	1.5292	1.5881	Sodium/calcium exchanger NCL2 [Glycine soja]

**Figure 6:**
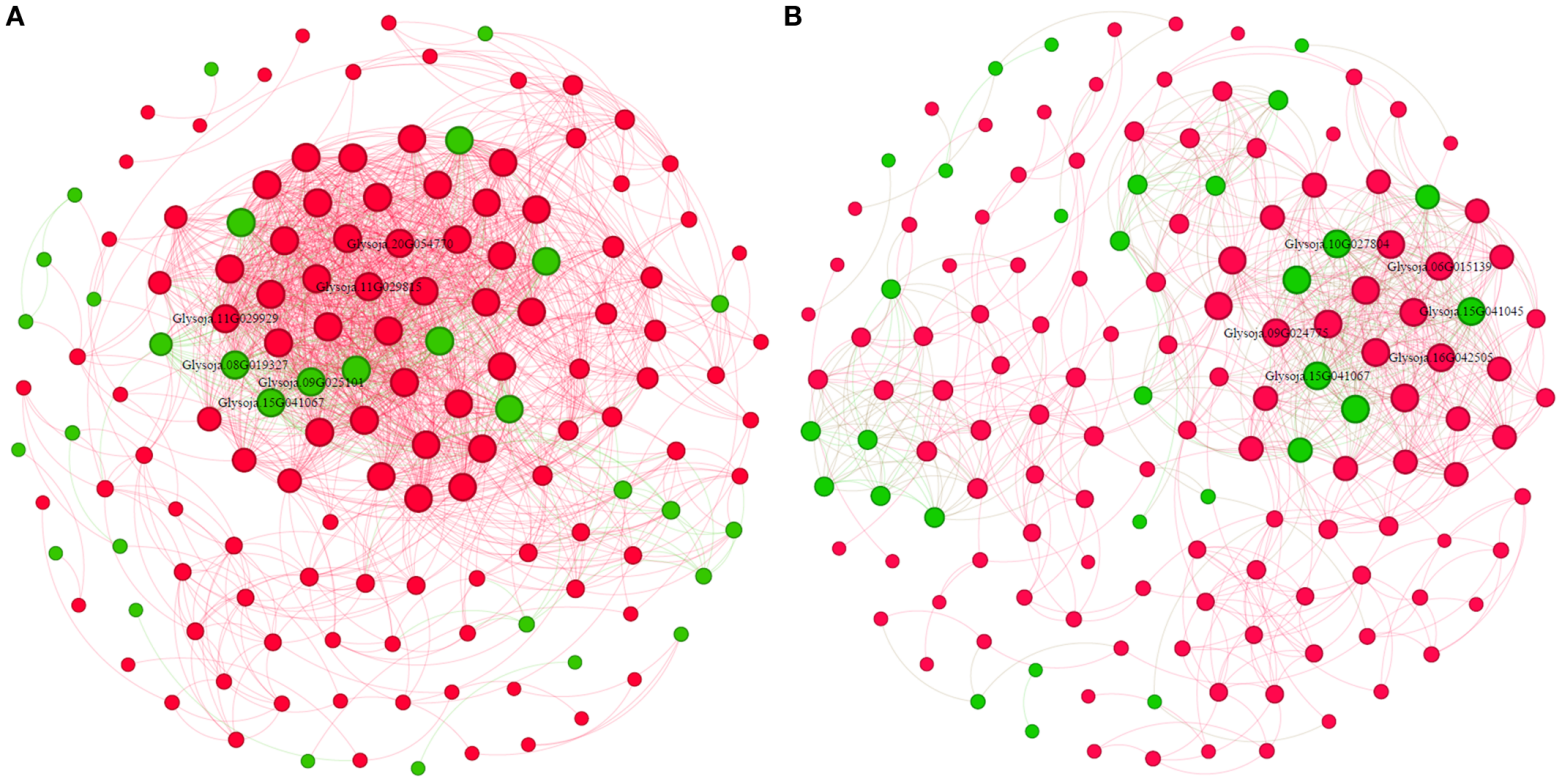
Co-expression network of transcription factors and Na^+^-related differentially expressed genes (DEGs) under **(A)** salt and **(B)** alkali stress. Green nodes indicate Na^+^ influx or storage genes; red nodes denote TFs.

Moreover, in total, 385 transcription factor (TF)-encoding DEGs were identified, predominantly from MYB (19%), ERF (17%), bHLH (17%), and WRKY (14%) families, collectively accounting for 67% of all TFs (**Supplementary Figure 3**). Co-expression network analysis revealed a more complex TF-gene regulatory interaction under salt stress than alkali stress (**Figure 6**). Salt-responsive TFs included *Glysoja.11G029929*, *Glysoja.11G029815*, and *Glysoja.20G054770*, whereas alkali-responsive TFs included *Glysoja.06G015139*, *Glysoja.09G024775*, and *Glysoja.16G042505*. Key Na^+^ transporter genes responsive under salt stress included *Glysoja.08G019327*, *Glysoja.15G041067*, and *Glysoja.09G025101*, while *Glysoja.10G027804*, *Glysoja.15G041045*, and *Glysoja.15G041067* were associated with alkali stress.

### 3.8 qRT-PCR validation

Six DEGs related to flavonoid biosynthesis or Na^+^ influx and storage were randomly selected for qRT-PCR analysis, using specific primers designed using Primer Premier 5.0 (**Supplementary Table 4**), to determine the mRNA levels after exposure to salt (0.6% NaCl and 1.2% NaCl) and alkali (pH 9.16) stress treatments,. The results showed that the relative gene expression trends of qRT-PCR were consistent with RNA-seq, demonstrating the reliability of the RNA-seq data (**Supplementary Figure 4**).

### 3.9 Flavonoids supplementation improves salt and alkali stress tolerance

**Figure 7:**
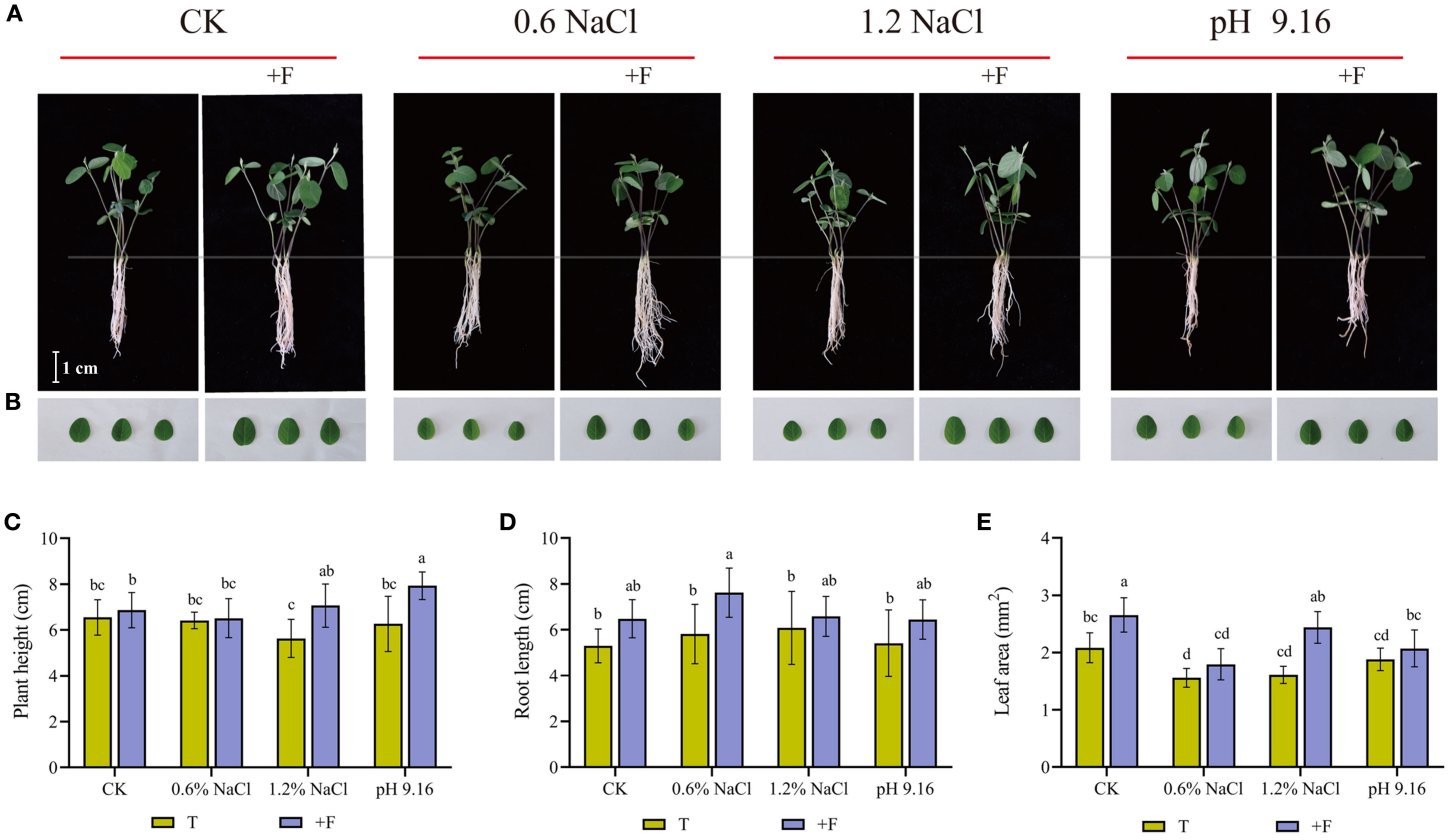
Assessment of morphological parameters in wild soybean under salt and alkali stress with and without flavonoid treatment. **(A)** Seedling morphology, **(B)** leaf appearance, **(C)** plant height, **(D)** root length, and **(E)** leaf area. Different lowercase letters denote statistically significant differences (ANOVA and Tukey’s *post hoc* test, *P < 0.05*).T, treatments without flavonoid; +F, treatments with flavonoid.

To further validate the functional relevance of flavonoids, exogenous applications of rutin and eriodictyol were conducted. Rutin (a flavonol glycoside) and eriodictyol (a central flavanone precursor) were selected due to their direct association with DEGs (*CYP75B1, F3’5’H, F6H*) in the flavonoid pathway. Under 1.2% NaCl and pH 9.16 treatments, flavonoid supplementation significantly increased plant height (**Figures 7A, C**). Root length was significantly enhanced under 0.6% NaCl stress, while leaf area showed a increase across all flavonoid-treated samples (**Figure 7B, E**). Collectively, these results demonstrate that flavonoids can enhance salt and alkali stress tolerance in wild soybean by promoting key growth parameters.

## 4 Discussion

Salt and alkali stress significantly hinder plant growth by disturbing ion equilibrium and inducing oxidative stress (Fang et al., 2021). A critical adaptive strategy observed in stress-tolerant plants involves the sequestration of excess Na^+^ in root tissues, thereby protecting photosynthetically active leaves (Wu et al., 2018). In this study, wild soybean germplasms showed significantly higher Na^+^ accumulation in roots than shoots under both salt and alkali stress. Na^+^ translocation was limited under alkali stress but more extensive under salt stress, indicating root-based Na^+^ sequestration as a key detoxification mechanism, particularly in high-pH environments (Jin et al., 2021). Transcriptomic analysis revealed that salt stress primarily upregulated Na^+^ sequestration-related genes, while alkali stress preferentially activated flavonoid biosynthesis pathways, suggesting a shift toward antioxidant and pH-buffering responses. These results underscore distinct stress-specific strategies in wild soybean, with root-localized ion retention and differential gene expression playing central roles in salinity and alkalinity tolerance.

Morphological observations revealed that wild soybean subjected to salt and alkali treatments exhibited suppressed seed germination and impaired seedling growth, with more pronounced inhibition under alkali stress. This is consistent with osmotic stress-induced growth restriction as previously reported (Li et al., 2025a). Although a slight reduction in plant height was observed, the difference was not statistically significant. These results suggested that alkali stress exerts a stronger inhibitory effect on early developmental stages than salt stress, likely due to the combined effects of high pH and osmotic imbalance (Paz et al., 2012).

Ion profiling under salt stress revealed a progressive increase in Na^+^ from roots to leaves, with roots showing the highest accumulation. Under alkali stress, Na^+^ was largely confined to roots. K^+^ was predominantly enriched in stems across all salt treatments, followed by alkali stress. The K^+^/Na^+^ ratio declined significantly under salt stress, especially in roots. In contrast, alkali stress maintained K^+^/Na^+^ ratios close to control levels, with stems showing the highest buffering capacity. This tissue-specific ion partitioning reflects a key mechanism for maintaining ionic homeostasis under saline-alkaline stress, consistent with previous reports in soybean and cotton (*Gossypium hirsutum* L.) that highlight the importance of ion homeostasis in stress tolerance (Guo et al., 2020; Sun et al., 2021a; Cai et al., 2022). This pattern was further supported by bioaccumulation (BF) and translocation (TF) factors. BF values were highest in roots across all treatments, particularly under high salinity, indicating effective root-based Na^+^ sequestration, a trend consistent with previous studies in soybean and rice (Li et al., 2016; Wang et al., 2022a). TF values were elevated in stems under salt stress, reflecting active Na^+^ translocation, as similarly reported by Xue et al. (2022). In contrast, under alkali stress, TF remained low across all tissues, suggesting restricted ion mobility likely due to high pH-induced ion transport inhibition (Yang et al., 2024). These results confirm that wild soybean relies on root ion retention and limited translocation under alkali conditions, whereas salt stress permits greater Na^+^ mobility, reflecting distinct ion management strategies in response to different stress types.

To further elucidate the regulatory mechanisms underlying salt and alkali stress responses in wild soybean, transcriptomic profiling of root tissues was conducted. DEGs were grouped into five clusters via hierarchical clustering. Clusters I and IV showed elevated expression under control and alkali stress conditions and were significantly enriched in lipid metabolism, cellular catabolic processes, and flavonoid biosynthesis pathways. This suggests a shift toward antioxidant defense and metabolic adjustment to mitigate high-pH-induced oxidative and structural stress. Such responses are consistent with previous findings demonstrating the protective role of flavonoids and lipid remodeling under alkali stress conditions (Xu et al., 2021; Gao et al., 2022). Flavonoids, synthesized via the phenylpropanoid pathway, act as key antioxidants and modulators of osmotic stress responses (Liu et al., 2024; Wang et al., 2024). Under alkali stress, significant enrichment of phenylpropanoid and flavonoid biosynthesis pathways was observed, involving key genes such as *PAL*, *4CL*, *COMT*, *CAD*, *UGT72E*, and *POD*, which are associated with lignin and flavonoid accumulation, contributing to enhanced structural integrity and reactive oxygen species (ROS) scavenging (Chen et al., 2019; Yan et al., 2025) (Preisner et al., 2014; Han et al., 2021; Liang et al., 2022; Ma et al., 2023). This response reflects a common protective strategy in plants under high-pH conditions, as previously reported in soybean and rice (Shao et al., 2016; Lu et al., 2022). Collectively, these findings highlight the central role of secondary metabolism in reinforcing alkali stress tolerance in wild soybean. In contrast, Cluster III was specifically upregulated under salt stress and enriched in plant hormone signal transduction and ABC transporter pathways, indicating an active role of transmembrane transport and hormone-mediated regulation in salinity adaptation. This is supported by earlier reports highlighting the involvement of ABC transporters and hormone crosstalk in salt tolerance mechanisms (Wang et al., 2023; Sahakyan and Sahakyan, 2025). The transcriptome analysis also revealed upregulation of genes involved in ion transport, particularly Na^+^/H^+^ antiporters and high-affinity K^+^ transporters, supporting their established roles in ionic detoxification and homeostasis in soybean (Sun et al., 2021b). While Clusters I and IV were enriched in genes related to secondary metabolism and oxidative defense under alkali stress, Cluster III showed predominant expression of ion transporters under salt treatment, highlighting a transcriptional divergence between stress types. These findings suggest that wild soybean roots coordinate distinct molecular responses, limiting Na^+^ mobility and enhancing ROS defense under alkali stress and activating ion transport mechanisms under salinity. This stress-specific partitioning of transcriptional programs underscores the adaptive flexibility of wild soybean to contrasting soil conditions.

Co-expression network analysis between DEGs and TFs revealed significant upregulation of key Na^+^ transport and homeostasis-related genes under salt stress. These included *HKT1* (*Glysoja.01G000023*), Na^+^/H^+^ antiporters, and H^+^ *-ATPases*, which facilitate cytosolic Na^+^ exclusion and vacuolar compartmentalization, an essential mechanism for mitigating ion toxicity (Horie et al., 2009; Yang and Guo, 2018; Wang et al., 2022b). Salt stress enhances transmembrane electrochemical gradients, facilitating Na^+^ influx into root cells via non-selective cation channels (NSCCs), cyclic nucleotide-gated channels (CNGCs), glutamate receptors (GLRs), and high-affinity K^+^ transporters (HKTs) (Demidchik and Maathuis, 2007; Keisham et al., 2018). Excess Na^+^ disrupts ion homeostasis, triggering responses to maintain Na^+^/K^+^ ratios (Munns and Tester, 2008; Jia et al., 2017). *HKT1* transporters play a central role in regulating long-distance Na^+^ transport and limiting root-to-shoot translocation. Decreased VcHKT1;1 transcript levels in blueberry plants led to increased Na^+^ concentrations in xylem sap and higher leaf Na^+^ contents compared with wild-type plants, indicating that VcHKT1;1 promotes leaf Na^+^ exclusion by retrieving Na^+^ from xylem sap (Song et al., 2023). In addition to xylem Na^+^ unloading, ScHKT1;2 was reported to be involved in Na^+^ uploading into the phloem, promoting Na^+^ recirculation from aerial parts to the roots in tomato (Romero-Aranda et al., 2021). The salt-specific upregulation of *HKT1* in this study suggests its involvement in root-localized Na^+^ retention in wild soybean. TFs enriched in the MYB, ERF, bHLH, and WRKY families were co-expressed with Na^+^ transporters under salt stress and with flavonoid biosynthesis genes under alkali stress, consistent with their established roles in ion homeostasis and oxidative stress responses (Wang et al., 2023). Notably, the regulatory networks under salt stress were more complex than those under alkali stress, suggesting that salt stress elicits a broader transcriptional reprogramming involving both ion transport and regulatory pathways. This highlights the distinct and dynamic coordination of TFs and transporters in modulating soybean responses to different types of ionic stress.

Exogenous application of flavonoids (rutin and eriodictyol) enhanced tolerance to both salt and alkali stress, validating their functional relevance. The selection of rutin and eriodictyol for exogenous flavonoid supplementation in soybean was based on their central roles in the flavonoid biosynthetic pathway and their involvement in stress responses (Gautam et al., 2023). Rutin, a flavonol glycoside, and eriodictyol, a core flavanone, are directly or indirectly synthesized and co-expressed through the activity of key genes such as flavonoid 3’-hydroxylase (CYP75B1, F3’H), which converts naringenin to eriodictyol, and flavonoid 3’,5’-hydroxylase (CYP75A, F3’5’H), which hydroxylates the B-ring of flavonoids (Forouzanfar et al., 2025; Li et al., 2025b; Liu et al., 2025). Under salt stress, upregulation of these genes enhances flavonoid accumulation, promoting reactive oxygen species (ROS) scavenging and improving plant tolerance (Li et al., 2025b; Wu et al., 2025). Treated wild soybean exhibited increased plant height under alkali stress, longer roots under both stresses, and larger leaf area under salt stress, indicating stress-specific improvements in growth. These findings align with previous reports linking flavonoid accumulation to enhanced salt tolerance in legumes (Praxedes et al., 2010; Duan et al., 2025; Wu et al., 2025).

In summary, wild soybean exhibited distinct transcriptional responses to salt and alkali stress. Salt stress primarily upregulated genes related to Na^+^ transport and homeostasis, along with co-expressed stress-responsive TFs, promoting root-based Na^+^ retention and ionic balance. Conversely, alkali stress induced flavonoid biosynthetic genes, likely enhancing antioxidant defenses against high-pH and bicarbonate-induced oxidative stress. This was supported by exogenous flavonoid supplementation, which mitigated growth inhibition, particularly under alkali conditions. These findings reveal stress-specific adaptive mechanisms and identify molecular targets for enhancing legume tolerance to saline-alkaline environments.

## 5 Conclusion

In conclusion, our findings demonstrate that wild soybean employs distinct molecular strategies to cope with salt and alkali stress. Salt stress primarily activates ion transport and homeostasis pathways to maintain cytosolic Na^+^/K^+^ balance, whereas alkali stress predominantly induces flavonoid biosynthesis to counteract oxidative damage under elevated pH conditions. The enhanced tolerance observed with exogenous flavonoid application further confirms their functional role in alkali stress mitigation. These insights not only deepen our understanding of stress-specific plant responses but also provide valuable targets for breeding salt-alkali resilient soybean cultivars.

## Data Availability

The datasets presented in this study can be found in online repositories. The names of the repository/repositories and accession number(s) can be found in the article/**Supplementary Material**.

## Supplementary material

Supplementary Figure 1 Phenotype of wild soybean at seed germination stage under normal, salt stress (0.6% NaCl and 1.2% NaCl) and alkali stress (pH 9.16 and pH 10.83) treatments. Thirty seeds were used for each treatment, and three replicates were performed for each treatment.

Supplementary Figure 2 Phenotype of seedling growth of wild soybean under normal, salt stress (0.6% NaCl and 1.2% NaCl) and alkali stress (pH 9.16 and pH 10.83) treatments for three and seven days.

Supplementary Figure 3 Types and proportion of differentially expressed transcription factors.

Supplementary Figure 4 Validation of RNA-Seq results using qRT-PCR. Comparing the log_2_FC values of RNA-seq and qRT-PCR (log_2_FC ≥ 1 indicates upregulation, log_2_FC ≤ 1 indicates downregulation). The gray bar charts show the results of qRT-PCR, and the line graphs show the results of RNA-seq. Error bars represent the SD of the mean(*n* = 3).

Supplementary Table 1 Non-redundant unigenes annotated in the transcriptome analysis.

Supplementary Table 2 Different expressed genes between different treatments.

Supplementary Table 3 Different expressed genes related to phenylpropanoid and flavonoid biosynthesis under salt and alkali stress treatments.

Supplementary Table 4 Primers for qRT-PCR.
