# Supplementary figures and images for: Root-centered sodium sequestration and transcriptomic regulation under salt and alkali stress in wild soybean (Glycine soja)
Source: Front Plant Sci. 2025 Sep 18;16:1675559. doi: 10.3389/fpls.2025.1675559 (PMC12488681; doi:10.3389/fpls.2025.1675559)

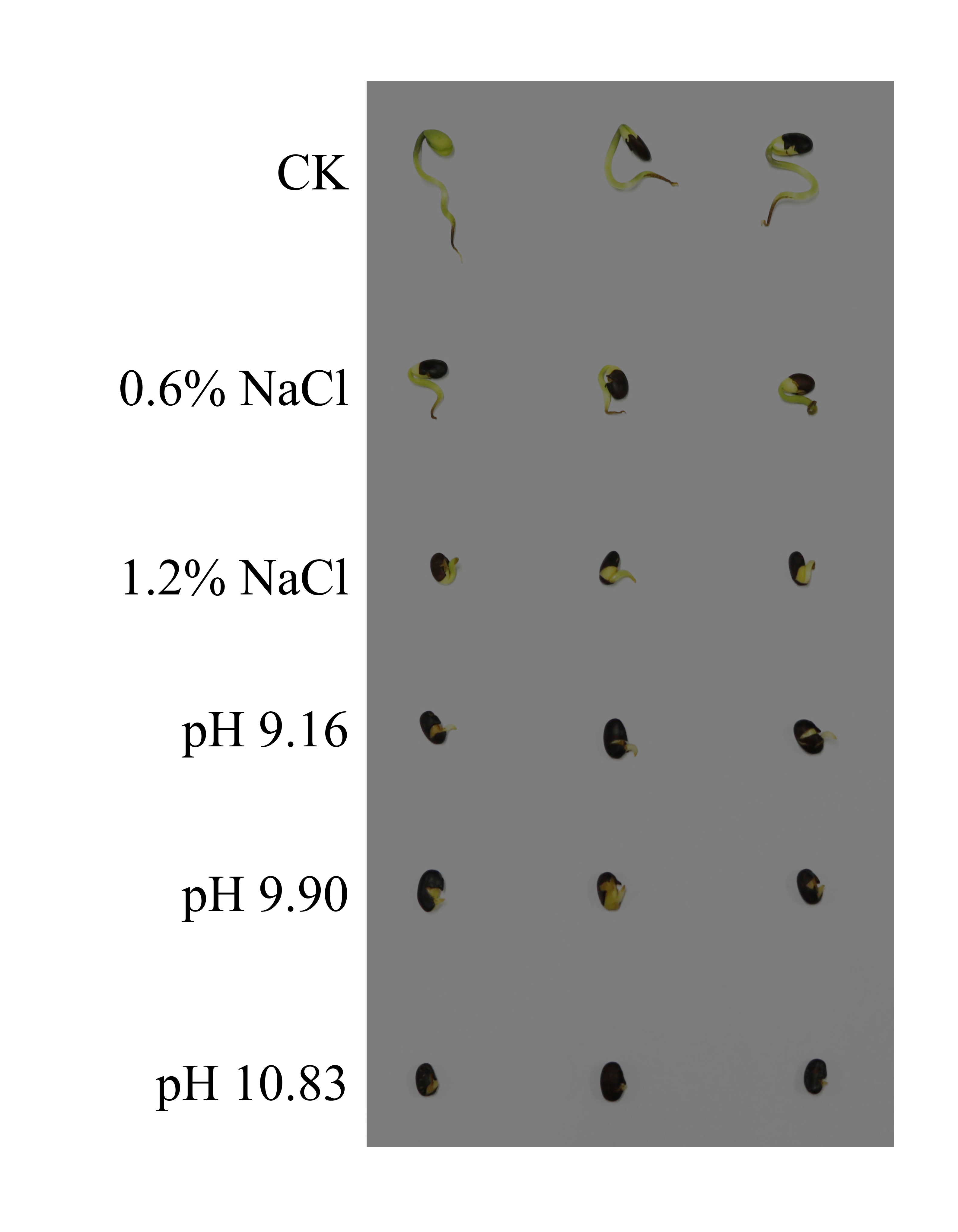

Supplement: Supplementary Figure 1 — Phenotype of wild soybean at seed germination stage under normal, salt stress (0.6% NaCl and 1.2% NaCl) and alkali stress (pH 9.16 and pH 10.83) treatments. Thirty seeds were used for each treatment, and three replicates were performed for each treatment. [file Image1.tif]

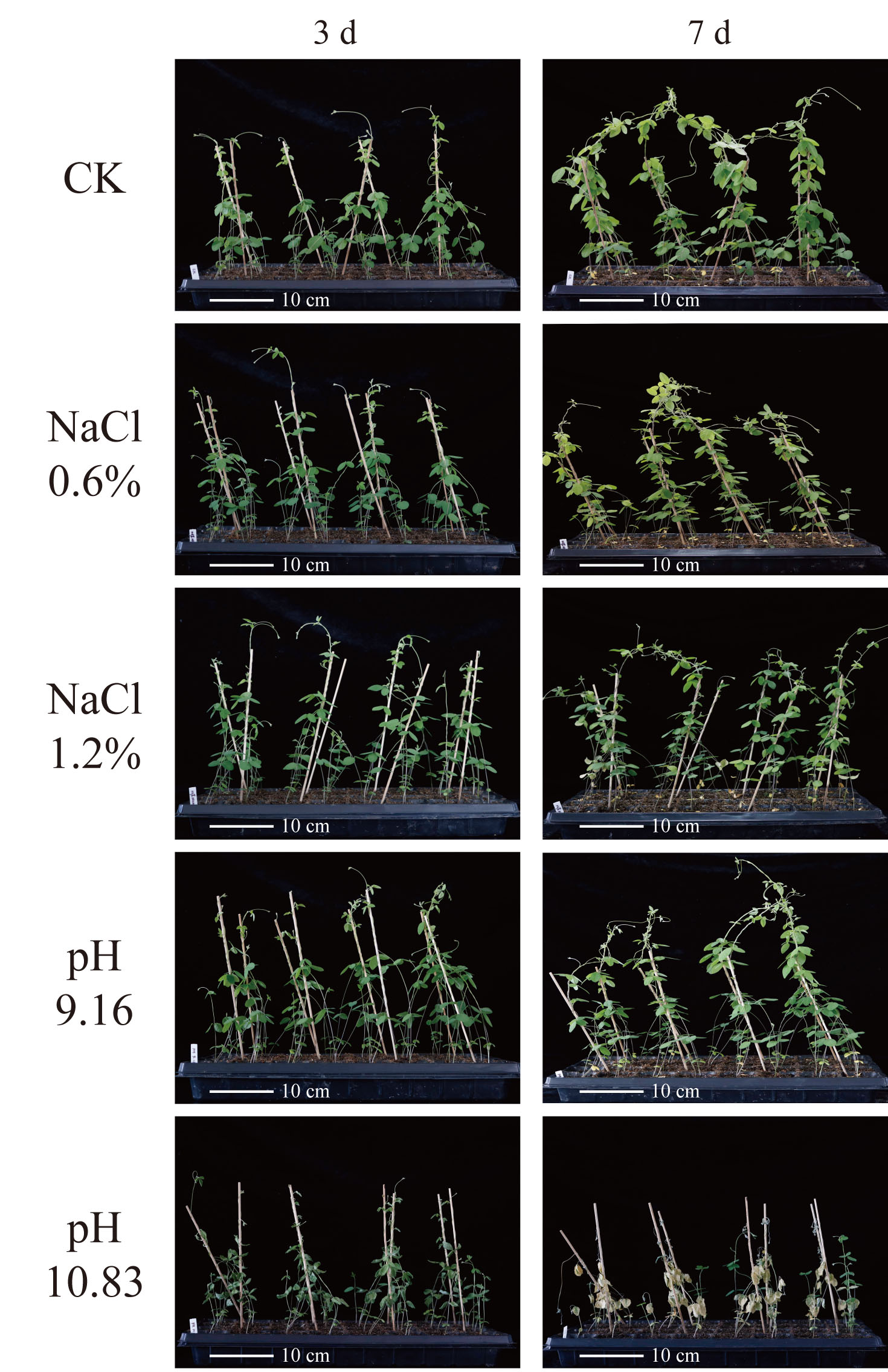

Supplement: Supplementary Figure 2 — Phenotype of seedling growth of wild soybean under normal, salt stress (0.6% NaCl and 1.2% NaCl) and alkali stress (pH 9.16 and pH 10.83) treatments for three and seven days. [file Image2.jpeg]

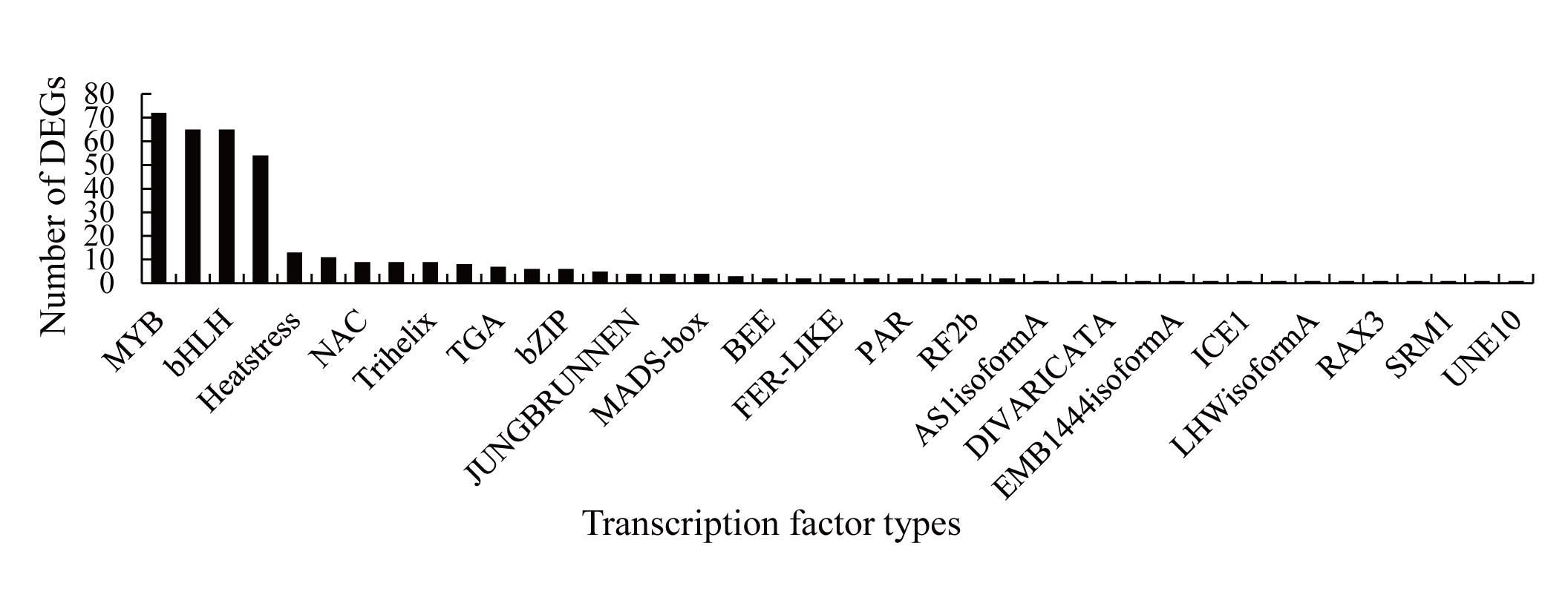

Supplement: Supplementary Figure 3 — Types and proportion of differentially expressed transcription factors. [file Image3.tif]

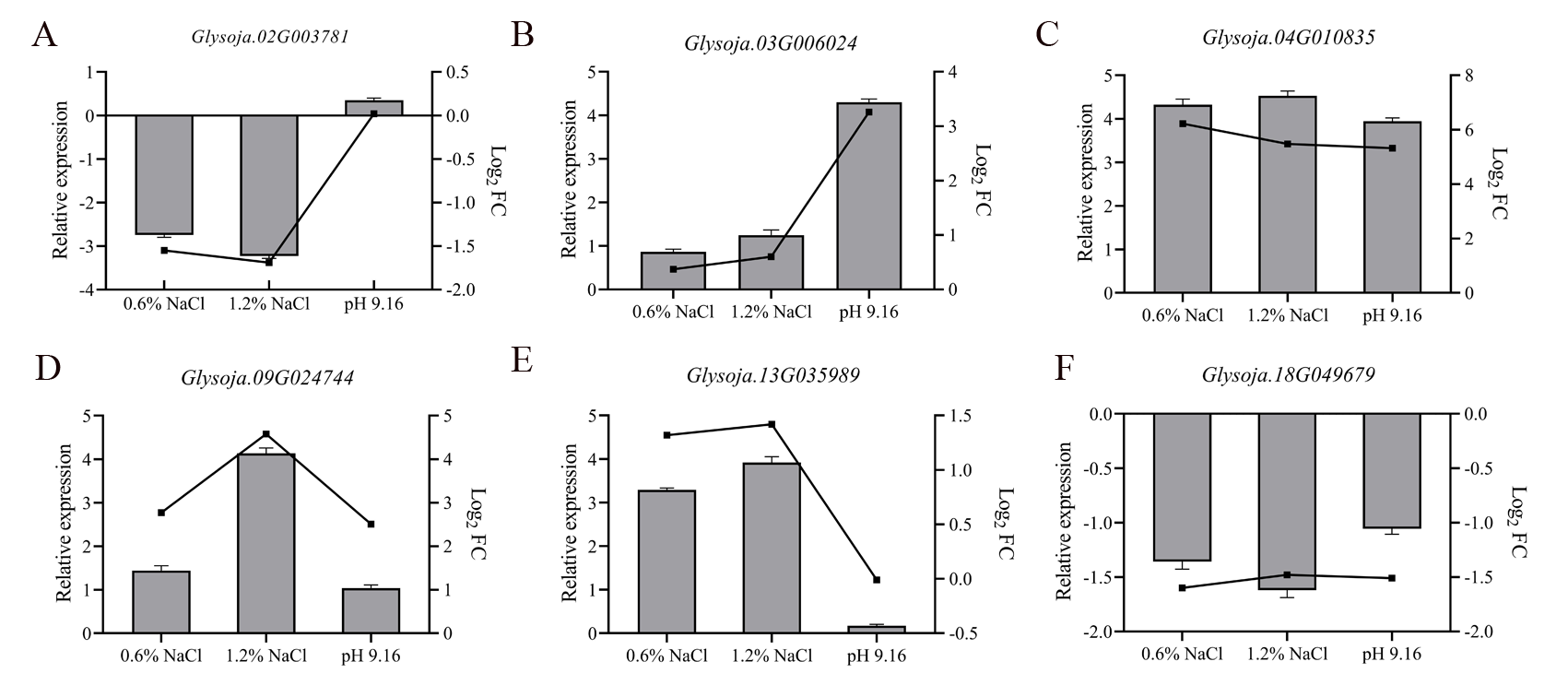

Supplement: Supplementary Figure 4 — Validation of RNA-Seq results using qRT-PCR. Comparing the log2FC values of RNA-seq and qRT-PCR (log2FC ≥ 1 indicates upregulation, log2FC ≤ 1 indicates downregulation). The gray bar charts show the results of qRT-PCR, and the line graphs show the results of RNA-seq. Error bars represent the SD of the mean(n = 3). [file Image4.tif]
